# Parental first aid literacy in epistaxis: do parents know what to do?

**DOI:** 10.3389/fpubh.2025.1551197

**Published:** 2025-04-30

**Authors:** Alya AlZabin, Ebtesam Almajed, Reem AlTamimi, Nuwayyir Abdullah Alqasimi, Yasser Albalawi, Nasser AlWehaibi

**Affiliations:** ^1^College of Medicine, Princess Nourah bint Abdulrahman University, Riyadh, Saudi Arabia; ^2^Department of Otolaryngology-Head and Neck Surgery, King Abdullah bin Abdulaziz University Hospital, Riyadh, Saudi Arabia

**Keywords:** epistaxis, nosebleed, first aid, parents, otorhinolaryngology, Saudi Arabia

## Abstract

**Background:**

Epistaxis is a common otorhinolaryngology emergency, especially among the pediatric population. While prompt first aid is essential, there is limited data on parents’ knowledge regarding its management in Saudi Arabia. This study assessed parental literacy in epistaxis first aid and its correlation with sociodemographic factors.

**Methods:**

A cross-sectional online study was conducted from May to June 2023, targeting parents of children aged ≤12 years across Saudi Arabia. Using a structured questionnaire, data on demographic characteristics and epistaxis first aid knowledge were collected from 777 participants. Descriptive statistics and binary logistic regression were utilized for analysis.

**Results:**

The majority of parents (79%) reported that their child had experienced epistaxis, and 72.7% considered it an emergency. However, knowledge gaps were evident: only 15.8% recognized posterior bleeds as life-threatening, and just 54.8% identified the correct position for managing epistaxis. Geographic disparities were noted, with parents in the South, East, and North showing lower literacy than those in the Western region. Additionally, male, non-Saudi, and postgraduate parents had reduced literacy regarding epistaxis prevention. A large proportion of parents relied on the internet for information, which may contribute to these gaps.

**Conclusion:**

There is a significant need for targeted educational programs to improve parental knowledge of epistaxis first aid, particularly among specific demographic groups. Enhancing access to first-aid training and credible resources could improve emergency responses to epistaxis, mitigating risks and promoting safer outcomes for children.

## 1 Introduction

Epistaxis is defined as bleeding from the nasal cavity or the nasopharynx. Among pediatric patients, epistaxis is one of the most common acute otorhinolaryngology emergencies; the incidence of epistaxis is rare during the first 2 years of life, but it increases rapidly afterward (1). Epistaxis can be classified as anterior or posterior epistaxis. Anterior epistaxis occurs more frequently. In contrast, posterior epistaxis is less common (2).

As an urgent medical emergency, epistaxis requires first aid to reduce pain and prevent deterioration (3). The primary objectives of a first aid provider are to preserve life, alleviate suffering, prevent further illness or injury, and to promote recovery (4). Per guidelines issued by the Ministry of Health (MOH) of Saudi Arabia, epistaxis first aid involves holding the lower portion (cartilage) of the nose on both sides while bending forward. Ideally, the pressure should be applied for at least 5 min for children and 10 to 15 min for adults. A cold pack may be applied if necessary. Emergency medical attention should be sought if the bleeding persists for more than 30 min (5).

Children are at risk for developing epistaxis spontaneously and due to trauma. However, most parents may not be familiar with the proper first aid management of epistaxis. A recent study led in 2021 conducted in AlQassim region focused explicitly on mothers’ knowledge of first aid measures. The findings of this study highlighted that only 38% of mothers knew how to help a child who had a nosebleed (6). Another cross-sectional study investigated the knowledge and practice of first aid among parents attending primary health care centers in Madinah city in Saudi Arabia. According to the study results, only 27.9% of the 390 parents correctly answered the question regarding how to manage a case of epistaxis (7). In Buraidah City, Saudi Arabia, Bassam (8) conducted a study to evaluate maternal knowledge and attitudes regarding first aid. The study sample consisted of 1,000 mothers. The study results indicated that less than half (45%) of mothers knew the correct epistaxis first aid (8). A quasi-experimental study evaluated how educational guidelines affect mothers’ performance regarding care for children with epistaxis. According to this study, 68.6% of mothers had unsatisfactory practices regarding epistaxis pre-educational guidelines (9).

To our knowledge, no nationwide study has been conducted on parents’ knowledge of epistaxis first aid in Saudi Arabia. Despite being common, there is a lack of general awareness of how to treat this condition among parents. Most of a child’s time is spent at home with their parents. A child’s curiosity makes them susceptible to unintentional injuries. If parents intervene quickly and correctly, they can limit the severity of the child’s injuries. Herein, this study aims to assess the literacy of parents in Saudi Arabia regarding epistaxis first aid and pediatric epistaxis and whether literacy score correlates with different sociodemographic factors.

## 2 Materials and methods

The study was conducted according to the guidelines of the Declaration of Helsinki and approved by the Institutional Review Board at Princess Nourah bint Abdulrahman University (PNU) (IRB log number: 23-0323). All participants were provided with informed consent prior to the commencement of the study. The research objectives were explained to all participants, and it was clarified that they could withdraw from the study without any consequences.

### 2.1 Sample and data collection

An online-based cross-sectional study with national coverage was conducted in Saudi Arabia. Saudi Arabia is the largest country in the Arabian Peninsula, spans 2.15 million square kilometers. It has five major regions (Central, Western, Eastern, Northern, and Southern) and a population of around 36 million as of 2024. The country has a well-developed healthcare system with both public and private sectors. Study participants were recruited from the five major regions and data collectors who resided in the corresponding region distributed the survey to ensure a representative sample. We used convenience sampling, distributing the survey through social media (X, WhatsApp, and Instagram) and parenting groups. The study was conducted from May 2023 to June 2023. The Inclusion criteria included (1) Parents aged 18 and older with children aged ≤ 12 years, (2) currently residing in Saudi Arabia, and (3) agreed to participate and complete the questionnaire. No gender restrictions were applied. The exclusion criteria included (1) individuals who did not consent to participate and (2) participants who did not complete the questionnaire. The sample size was estimated based on a power of 80% with a margin error of 5% and a confidence level of 95%; the minimum sample size required was 610 participants, as computed by G-power software. The total sample size comprised 777 participants; additional responses were accepted to account for potential incomplete responses and ensure robust statistical analysis across different subgroups.

### 2.2 Study survey

The study employed a self-administered questionnaire modified from previous studies in alignment with the study’s objectives (2, 10–12). All data were obtained and managed using Google Forms electronic tool. The questionnaire was reviewed first by a clinical expert on the subject and an emergency consultant then was piloted on a cohort of non-medical people. Data was collected from participants using a questionnaire consisting of two sections. (1) Sociodemographic data questions included age, nationality, current residency, marital status, highest educational level, occupation status, Household Income (HHI), number of children, and the age of the oldest and youngest child. (2) Parents’ literacy toward epistaxis first aid and its prevention.

### 2.3 Statistical analysis

A comprehensive statistical analysis was conducted on the dataset, encompassing both descriptive and inferential methodologies. Firstly, a descriptive analysis was conducted to summarize the demographic characteristics of the participants, which included age, gender, and other features. This provided an overview of the study population. Subsequently, the Binary Logistic Regression Model was employed to determine the predictors of high literacy of Epistaxis First Aid and Prevention in Saudi Arabia. Statistical significance was established at a *p*-value of 0.05 or lower and a 95% Confidence Interval (CI). All statistical analyses were executed using IBM’s SPSS Software, version 29.0.

## 3 Results

Seven hundred seventy-seven parents residing in Saudi Arabia enrolled themselves electively and completed the study questionnaire. The participants’ sociodemographic characteristics are displayed in Table 1. Notably, the majority were mothers (85.6%, *n* = 665), while fathers constituted 14.4% (*n* = 112). The average age was 37.2 years (SD = 8.4), with a range of 19 to 67 years. Our sample predominantly comprised Saudi parents (91.4%, *n* = 710) and married participants (93.7%, *n* = 728). Geographically, the Western Region had the highest representation (31.1%, *n* = 242). Educationally, 65.0% (*n* = 505) held a bachelor’s degree, and most were employed (48.8%, *n* = 379). Monthly income varied, with 52.3% (*n* = 406) earning 5,000–10,000 SAR. The median number of children was 3, with an average age of 12.4 years (SD = 7.4) for the oldest child and 5.1 years (SD = 3.5) for the youngest.

**Table 1:** Descriptive analysis of the participants sociodemographic characteristics (*n* = 777).

Variable		*N*	%
Gender	Mother	665	85.6
	Father	112	14.4
Age	Mean (SD)	37.2 (8.4)	
	Range	19–67	
Nationality	Non-Saudi	67	8.6
	Saudi	710	91.4
Marital status	Married	728	93.7
	Divorce/Widowed	49	6.3
Current residency	Western Region	242	31.1
	Central Region	156	20.1
	South Region	152	19.6
	Eastern Region	116	14.9
	North Region	111	14.3
Educational status	School	192	24.7
	Bachelors	505	65
	Post-Graduate Degree	80	10.3
Occupational status	Unemployed	42	5.4
	Employee	379	48.8
	Housewife	254	32.7
	Student	60	7.7
	Retired	42	5.4
Monthly income	< 5,000 SAR	164	21.1
	5,000–10,000 SAR	406	52.3
	>10,000 SAR	207	26.6
Number of children	Median	3	100
	Range	1–10	
Age of oldest child	Mean (SD)	12.4 (7.4)	
	Range	1–31	
Age of youngest child	Mean (SD)	5.1 (3.5)	
	Range	1–12	

Table 2 shows the assessment of parents’ literacy toward epistaxis first aid. The majority (79%, n = 614) had heard, witnessed, or experienced nosebleeds. A significant portion (72.7%, *n* = 565) considered nosebleeds emergencies. Regarding training, 47% (*n* = 365) had received first-aid management awareness. While 96.3% (*n* = 748) correctly identified epistaxis as bleeding from the nose. Among the types of nosebleeds, 15.8% (*n* = 123) recognized posterior bleeds as potentially life-threatening, while a significant portion (69.1%, *n* = 537) admitted uncertainty. Concerning the duration of nose pinching, 30.1% (*n* = 234) recommended at least 10–15 min. In the context of children, 55% (*n* = 427) advocated pinching for at least 5 min. The understanding of the nose’s anatomical regions to be pinched varied, with 33.6% (*n* = 261) answered with bony part, 33.7% (*n* = 262) of the participants choose the cartilage part, and 17.4% (*n* = 135) of both. Notably, 71.4% (*n* = 555) recognized that breathing should be allowed through the mouth during nose pinching. Regarding the patient’s position, 54.8% (*n* = 426) recommended sitting with the head tilted forward, while 24.1% (*n* = 187) suggested sitting with the head tilted backward. Notably, 10.8% (*n* = 84) were uncertain about the proper position. Regarding the use of ice cubes 29.7% (*n* = 231) believed that sucking an ice cube could help reduce nose bleeding. Similarly, there was uncertainty about the efficacy of icing on the neck region, with 14.9% (*n* = 116) believing it could help. In contrast, a majority (59.3%, *n* = 461) believed that cooling or icing on the nose could indeed slow blood flow. In the context of managing a child’s nosebleed, 64% (*n* = 497) recommended pinching the nose, while various alternative methods were suggested by 10.9% (*n* = 85) of participants.

**Table 2:** Assessment of parents’ literacy toward epistaxis first aid.

Survey item		*N*	%
Have you ever heard/witnessed/experienced nosebleeds?	No	163	21
	Yes	614	79
Do you consider nosebleeds as one of the emergencies?	No	212	27.3
	Yes	565	72.7
Have you ever received any training or awareness program on first-aid management?	No	412	53
	Yes	365	47
Epistaxis is Bleeding from	I do not know	16	2.1
	Nose	748	96.3
	Ear	13	1.7
Which of the following can be a life-threatening nosebleed?	Anterior	79	10.2
	Lateral	17	2.2
	Medial	21	2.7
	Posterior	123	15.8
	I do not know	537	69.1
For how long shall nose pinching be performed? (mins)	At least 10 to 15 min	234	30.1
	At least 20 min	15	1.9
	At least 5 min	384	49.4
	I do not know	144	18.5
For how long shall nose pinching in children be performed? (mins)	At least 10 to 15 min	144	18.5
	At least 20 min	26	3.3
	At least 5 min	427	55
	I do not know	180	23.2
Which part of the nose shall be pinched with fingers to stop nose bleeding?	Bony (upper part)	261	33.6
	Cartilage (lower part)	262	33.7
	Both	135	17.4
	I do not know	119	15.3
What care should be taken for breathing during nose pinching? Breathing shall be	Allowed through mouth avoiding blood swallowing	555	71.4
	Halted during nose pinching	20	2.6
	Maintained through oxygen pump	19	2.4
	I do not know	183	23.6
What is the proper position that a patient with epistaxis should take?	Lying down with ice pack over the nasal bridge	80	10.3
	Sitting with head tilted backward	187	24.1
	Sitting with head tilted forward	426	54.8
	I do not know	84	10.8
Do you think sucking an ice cube can help in reduction of nose bleeding?	No	231	29.7
	Yes	231	29.7
	I do not know	315	40.5
Do you think the icing on the neck region may help in the management of epistaxis?	No	282	36.3
	Yes	116	14.9
	I do not know	379	48.8
Do you think cooling or icing on the nose helps in slowing blood flow from the nose?	No	102	13.1
	Yes	461	59.3
	I do not know	214	27.5
If your child’s nose is bleeding, how would you stop it?	Obstruct nose with cotton	100	12.9
	Obstruct nose with tissue paper	95	12.2
	Pinching the nose	497	64
	Other method	85	10.9

**Figure 1:**
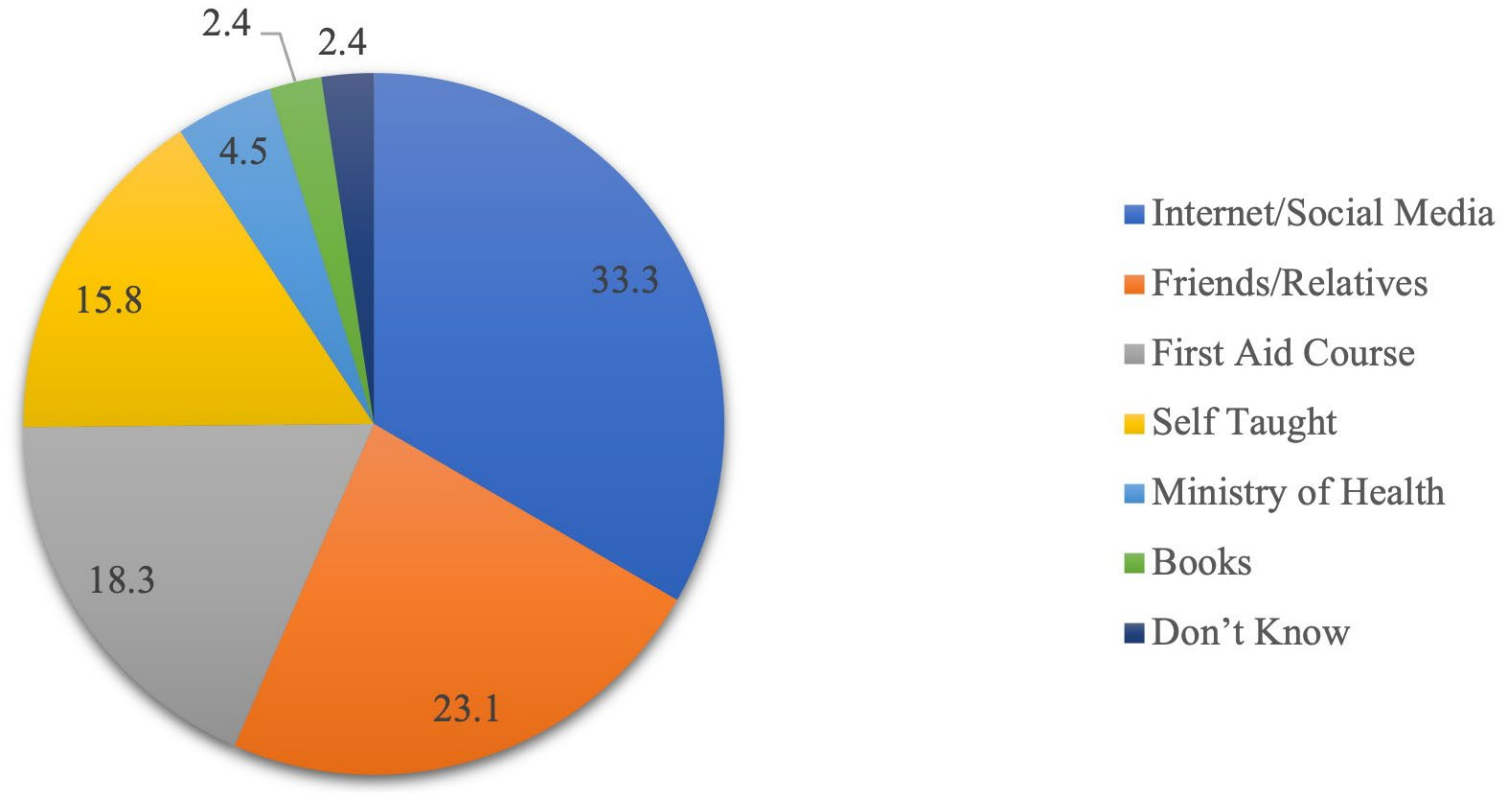
Knowledge about the source of information of epistaxis first aid.

Figure 1 shows the sources of information about epistaxis first aid among the participants. The majority (33.3%) reported obtaining knowledge from the Internet or social media. Friends and relatives accounted for 23.1%, indicating a significant influence from personal networks. First aid courses played a role in 18.3% of respondents’ knowledge acquisition, while self-teaching was a source for 15.8%. A smaller percentage (4.5%) mentioned the Ministry of Health as a source. Books contributed to the understanding of epistaxis first aid for 2.4%, and an equal percentage (2.4%) admitted not knowing the source of their information.

**Figure 2:**
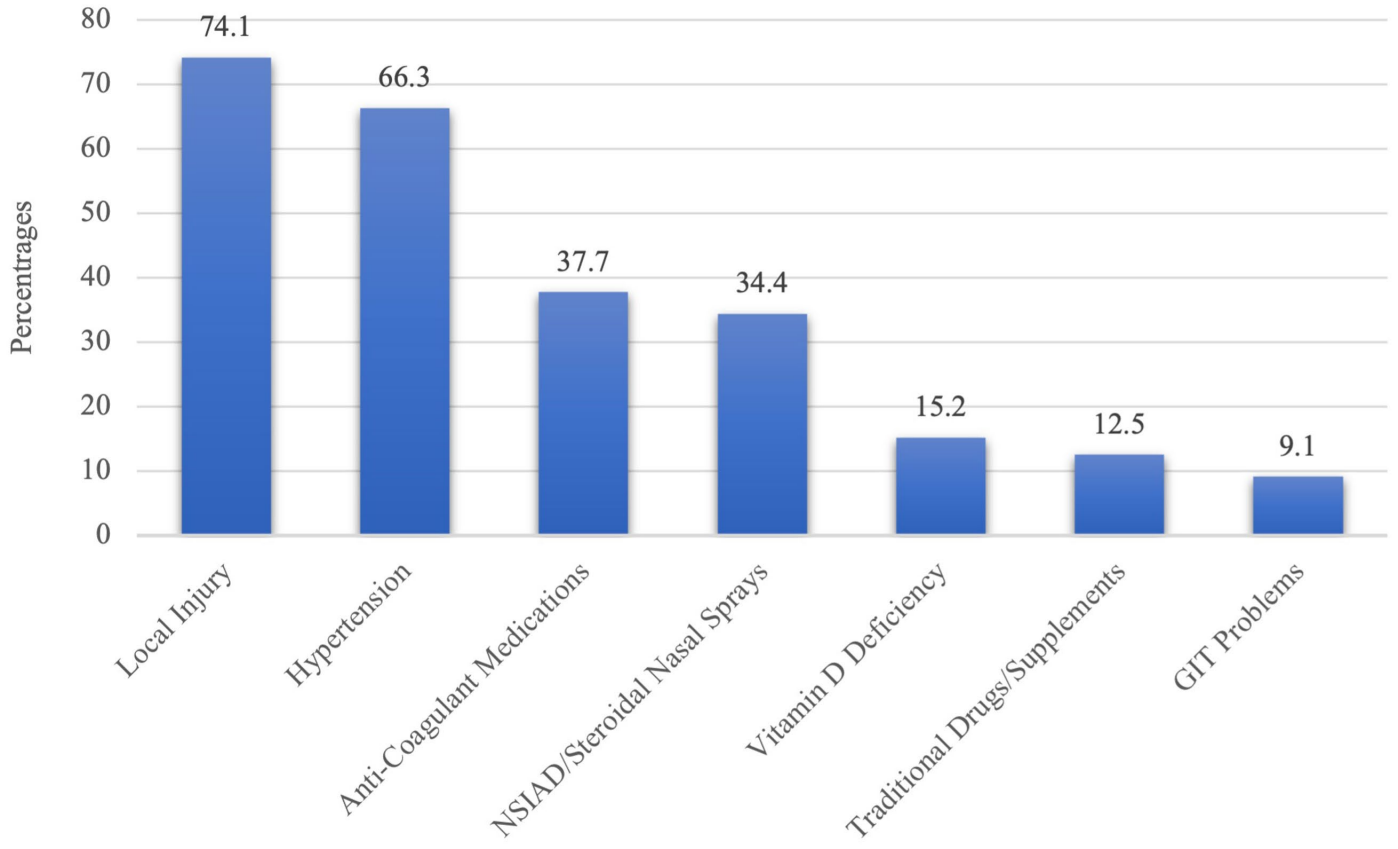
Knowledge about the risk factors of epistaxis.

Figure 2 shows participants’ knowledge about the risk factors of epistaxis. Local injury was identified by the majority, with 74.1% recognizing it as a risk factor. Hypertension was well-acknowledged, with 66.3% recognizing its association with epistaxis. Anti-coagulant medications were identified by 37.7% as a potential risk factor, and 34.4% recognized non-steroidal anti-inflammatory drugs (NSAIDs) or steroidal nasal sprays as contributing factors. Vitamin D deficiency was acknowledged by 15.2% of participants. Traditional drugs or supplements were recognized by 12.5%, while gastrointestinal (GIT) problems were identified by 9.1%.

Table 3 shows the knowledge hierarchy concerning when individuals perceive it necessary to seek emergency room (ER) care for epistaxis, or nosebleeds, based on their assigned ranks and corresponding mean scores. At the forefront is the recognition that severe bleeding causing breathing difficulty (dyspnea) demands the utmost urgency, securing the top position with a mean score of 4.54 and ranking first. Subsequently, respondents emphasize the significance of persistent bleeding even after performing initial first aid (mean 4.44, 2nd rank) and nosebleeds accompanied by chest pain or dizziness (mean 4.42, 3rd rank). The fourth position is assigned to situations where the person appears pale or fatigued (mean 4.38), indicating a perceived level of urgency. The fifth and sixth ranks pertain to scenarios involving frequent nosebleeds alongside bruises or bleeding from other places (mean 4.33) and bleeding after surgery close to the nose or in the presence of a nasal tumor (mean 4.32), respectively. Following these, the seventh and eighth ranks focus on situations where individuals taking anticoagulant medications experience unstoppable nosebleeds (mean 4.23) and bleeding occurs after a facial injury (mean 4.21), respectively.

**Table 3:** Knowledge about when to go ER in case of epistaxis in accordance to their rank.

Scenario to seek ER care	Mean (SD)	Mean rank
If the bleeding is severe, causing breathing difficulty (dyspnea)	4.54 (1.2)	1st
If the bleeding still does not stop even after performing first aid	4.44 (1.2)	2nd
If the nosebleeds are severe, and the person suffers chest pain, or feels dizzy	4.42 (1.2)	3rd
If the person appears pale or fatigued	4.38 (1.3)	4th
If the nosebleeds occur frequently, or when bruises appear in the body or bleeding from other places, while current bleeding does not stop	4.33 (1.3)	5th
If bleeding occurs after surgery close to the nose, or if the person has a nasal tumor	4.32 (1.3)	6th
If the person is taking anticoagulant medications, such as aspirin, clopidogrel, or warfarin, while nosebleeds do not stop	4.23 (1.3)	7th
If bleeding occurs after a facial injury	4.21 (1.4)	8th

**Figure 3:**
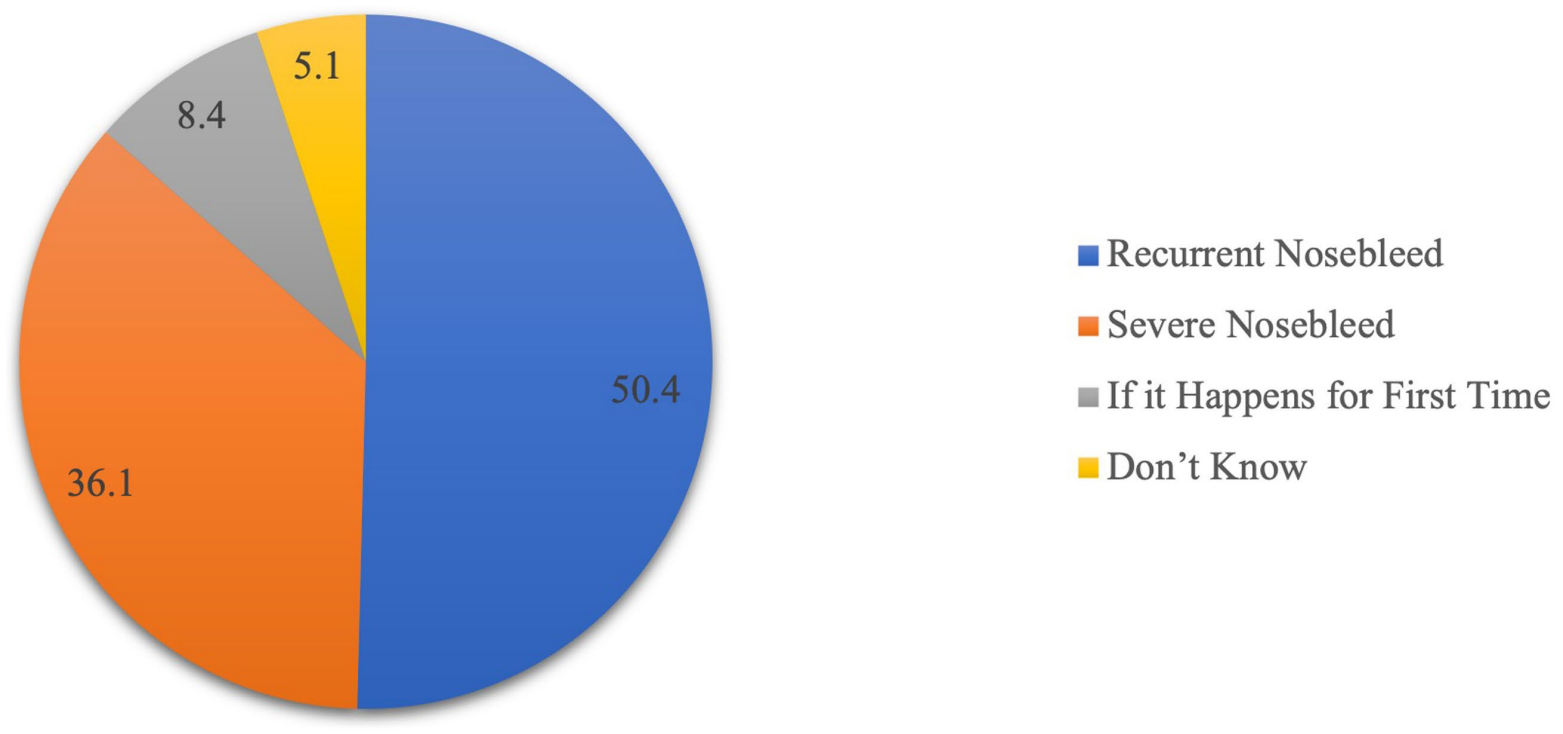
Participants’ awareness about the time of ENT physician consultation.

Figure 3 shows participants’ awareness regarding the appropriate times for consulting an Ear, Nose, and Throat (ENT) physician. Notably, 50.4% are aware that recurrent nosebleeds warrant consultation, suggesting a recognition of the importance of seeking medical advice for persistent issues. For severe nosebleeds, 36.1% of participants acknowledged the need for ENT consultation, indicating a reasonable understanding of the severity threshold. Only 8.4% recognized the importance of consulting an ENT physician if nose bleeding occurs for the first time, suggesting that a smaller proportion is aware of the significance of initial occurrences. Additionally, 5.1% expressed uncertainty about when to consult an ENT physician.

Table 4 shows parents’ literacy toward various preventive measures for epistaxis following an episode. Notably, 20.2% (*n* = 157) strongly agreed or agreed with the local application of bacitracin or petrolatum ointment, while 25.7% (*n* = 200) expressed agreement with the use of the nasal saline spray. A substantial 44.0% (*n* = 342) strongly agreed or agreed with humidifying the air with a cool mist vaporizer. The practice of sneezing gently with the mouth open received agreement from 41.3% (*n* = 321) of participants. The highest agreement was observed for avoiding digital trauma by keeping the child’s nails well-trimmed, with a significant 67.7% (*n* = 526) expressing approval. Avoiding strenuous activities garnered agreement from 58.3% (*n* = 453), while 47.4% (*n* = 368) agreed with avoiding hot showers. Additionally, 57.3% (*n* = 445) agreed with avoiding nose blowing and excessive sneezing, and 45.3% (*n* = 352) expressed agreement with avoiding hot and spicy foods while encouraging fluid intake.

**Table 4:** Assessment of parent’s literacy of epistaxis prevention following the episode of epistaxis.

Preventive measure		Strongly disagree/disagree	Neutral	Strongly agree/agree
Local application of bacitracin or petrolatum ointment	*N*	392	228	157
	%	50.5	29.3	20.2
Use nasal saline spray	*N*	307	270	200
	%	39.5	34.7	25.7
Humidifying the air with a cool mist vaporizer	*N*	180	255	342
	%	23.2	32.8	44.0
Should sneeze gently with the mouth open	*N*	184	272	321
	%	23.7	35.0	41.3
Avoid digital trauma (e.g., nose picking) by keeping the child’s nails well-trimmed	*N*	129	122	526
	%	16.6	15.7	67.7
Avoid strenuous activities	*N*	145	179	453
	%	18.7	23.0	58.3
Avoid hot showers	*N*	173	236	368
	%	22.3	30.4	47.4
Avoid nose blowing and excessive sneezing	*N*	136	196	445
	%	17.5	25.2	57.3
Avoid hot and spicy foods and drink plenty of fluids	*N*	191	234	352
	%	24.6	30.1	45.3

Table 5 shows the results of a Binary Logistics Regression analysis assessing the adjusted predictors of high parents’ literacy toward epistaxis first aid management. The variables considered include Gender, Age of Parents, Nationality, Current Residency, Marital Status, Highest Educational Level, Occupation Status, Household Income, Number of Children, Age of Oldest Child, and Age of Youngest Child. Among the significant predictors, the Current Residency region plays a notable role. Parents residing in the South (*B* = −0.961, *p* = 0.001), East (*B* = −0.488, *p* = 0.153), and North (*B* = −0.750, *p* = 0.027) regions exhibited diminished odds of possessing high literacy compared to their counterparts in the Western region. This geographical discrepancy underscores the influence of regional dynamics on parental knowledge in epistaxis first aid.

**Table 5:** Adjusted predictors of high parents’ literacy toward epistaxis first aid management (binary logistics regression).

Predictor variable	*B*	Sig.	aOR	95% CI	
				Lower	Upper
Gender (male)	−0.108	0.721	0.898	0.496	1.624
Age of parents	0.023	0.302	1.023	0.980	1.068
Nationality (Saudi)	0.081	0.829	1.084	0.521	2.258
Current Residency (Western)		0.027*			
Current Residency (Central)	−0.490	0.112	0.613	0.335	1.120
Current Residency (South)	−0.961	0.001*	0.382	0.213	0.685
Current Residency (Eastern)	−0.488	0.153	0.614	0.314	1.199
Current residency (North)	−0.750	0.027*	0.472	0.243	0.918
Marital status (divorced/widow)	0.036	0.934	1.037	0.441	2.438
Highest educational (post-graduate)	−0.257	0.188	0.774	0.528	1.133
Occupation status (unemployed)		0.775			
Occupation status (employee)	0.060	0.894	1.062	0.443	2.545
Occupation status (housewife)	−0.138	0.758	0.871	0.363	2.092
Occupation status (student)	0.395	0.463	1.485	0.516	4.271
Occupation status (retired)	0.173	0.810	1.189	0.291	4.861
Household income	0.193	0.247	1.213	0.875	1.682
Number of children	0.011	0.919	1.011	0.822	1.244
Age of oldest child	−0.016	0.638	0.984	0.920	1.053
Age of youngest child	0.031	0.492	1.032	0.944	1.129
Constant	1.299	0.184	3.667		

*Statistically significant.

Contrastingly, the occupation status of being a student (*B* = 0.395, *p* = 0.463) demonstrated a positive association with high literacy, although the lack of statistical significance suggests a need for cautious interpretation. The absence of a significant association may stem from various factors, including the diverse nature of student roles and responsibilities.

Moreover, intriguingly, several variables such as Gender, Age of Parents, Nationality, Marital Status, Highest Educational Level, Household Income, Number of Children, Age of Oldest Child, and Age of Youngest Child did not exhibit significant associations with high parents’ literacy in epistaxis first aid management. This implies that within this specific context, these factors may not be predominant influencers of parental literacy. In essence, this thorough examination sheds light on the multifaceted determinants of parental literacy in epistaxis first aid management, providing valuable insights for targeted interventions and educational initiatives.

Table 6 shows the outcomes of a Binary Logistics Regression examining adjusted predictors of high parents’ literacy regarding epistaxis prevention post-episode. Among the discerned predictors, several factors significantly impact parental literacy. Gender plays a pivotal role, with male parents demonstrating lower odds of exhibiting high literacy (*p* = 0.039, aOR = 0.626). This suggests a potential gender-based discrepancy in awareness and knowledge related to epistaxis prevention.

**Table 6:** Adjusted predictors of high parents’ literacy of epistaxis prevention following the episode of epistaxis (binary logistics regression).

Predictor variable	*B*	Sig.	aOR	95% CI	
				Lower	Upper
Gender (male)	−0.468	0.039*	0.626	0.402	0.976
Age of parents	−0.003	0.825	0.997	0.966	1.028
Nationality (Saudi)	−0.560	0.044*	0.571	0.331	0.985
Current residency (Western)		0.493			
Current residency (Central)	−0.300	0.157	0.741	0.489	1.123
Current residency (South)	−0.266	0.224	0.767	0.500	1.177
Current residency (Eastern)	−0.259	0.283	0.772	0.481	1.238
Current residency (North)	−0.006	0.981	0.994	0.613	1.612
Marital status (divorced/widow)	0.055	0.860	1.056	0.575	1.942
Highest educational (post-graduate)	−0.297	0.046*	0.743	0.555	0.995
Occupation status (unemployed)		0.012*			
Occupation status (employee)	0.314	0.362	1.369	0.696	2.692
Occupation status (housewife)	−0.342	0.327	0.710	0.359	1.407
Occupation status (student)	−0.432	0.307	0.649	0.283	1.488
Occupation status (retired)	0.193	0.690	1.212	0.470	3.127
Household income	0.043	0.727	1.044	0.820	1.329
Number of children	−0.018	0.812	0.982	0.847	1.139
Age of oldest child	0.006	0.805	1.006	0.959	1.055
Age of youngest child	−0.001	0.985	0.999	0.936	1.067
Constant	1.045	0.145	2.844		

*Statistically significant.

Nationality also emerges as a significant factor, with non-Saudi nationals exhibiting reduced odds of high literacy (*p* = 0.044, aOR = 0.571). This finding implies that cultural or contextual differences may influence the level of awareness and understanding among parents from different national backgrounds. Highest Educational Level proves to be another influential predictor, where parents with post-graduate education display lower odds of high literacy (*p* = 0.046, aOR = 0.743). This unexpected result challenges conventional assumptions and warrants further exploration into the dynamics of education and health literacy.

Interestingly, being unemployed is associated with increased odds of high literacy (*p* = 0.012), offering a unique perspective on the correlation between occupation status and epistaxis prevention knowledge. Other examined factors, including age, residency, marital status, occupation, income, and the number/ages of children, show no significant associations. These findings underscore the importance of considering diverse sociodemographic factors in tailoring educational interventions to enhance epistaxis prevention awareness among parents.

## 4 Discussion

To our knowledge, this is the first nationwide study in Saudi Arabia to assess parental knowledge of epistaxis first aid across all five major regions. Previous studies focused on specific cities or maternal knowledge, whereas our study provides a broader perspective by analyzing sociodemographic factors affecting first-aid literacy. Our study found that while 79% of parents had encountered epistaxis, significant knowledge gaps remained. Only 54.8% correctly identified the proper head position for nosebleed management, and just 15.8% recognized posterior bleeds as potentially life-threatening. Parental literacy varied by region, with lower knowledge levels in the South, East, and North. Additionally, non-Saudi parents and those with postgraduate education demonstrated reduced awareness of epistaxis prevention. These findings highlight the need for targeted educational programs to improve first-aid knowledge.

Epistaxis is one of the most common acute otorhinolaryngology emergencies. The incidence of epistaxis is rare during the first 2 years of life, but it increases rapidly afterward (1). Elsayed Elboraei et al. (13) reported a 47.4% occurrence rate. Controlling epistaxis and its complications, including blood loss, can severely impact a family’s quality of life (14). Merdad et al. (15) found that healthcare workers’ understanding of managing epistaxis was deficient, consequently affecting parental education. They observed a discrepancy between providers’ confidence and competence, which calls for better educational interventions. Consequently, this study assessed the literacy of first-aid management regarding epistaxis and the factors that could affect it among parents in Saudi Arabia.

In order to gain insight into the current state of knowledge regarding epistaxis first aid and to identify any gaps in the literature, an assessment of Saudi parents’ literacy regarding epistaxis first aid was conducted. In the present study, the high prevalence of reported hearing, witnessing, or experiencing epistaxis among participants (79%) may suggest a higher level of familiarity with first-aid management. However, the observed variability in understanding the correct location for applying pressure during nosebleeds and the fact that only 15.8% of participating parents correctly identified posterior nosebleeds as potentially life-threatening, in addition to over half of participants (54.8%) recommended an incorrect head position highlights a knowledge gap and contradicts our initial assumption. A thorough review of the literature by Alkhalifah et al. (16) identified 17 studies reporting 12,057 participants’ knowledge and attitudes regarding epistaxis in Saudi Arabia. According to this systematic review and meta-analysis, there are significant differences in epistaxis awareness levels based on factors such as age, gender, and geographical location. It was found that general Saudi populations and self-administered questionnaires had higher awareness levels. An estimated 63% of Saudi residents are aware of epistaxis and its management. Regarding parents’ knowledge of the risk factors of epistaxis, the majority correctly identified local injury as a primary risk factor, with 74.1% of respondents acknowledging its relevance, which was consistent with the literature (17). Hypertension was also widely recognized, with 66.3% understanding its potential association with epistaxis, aligning with established medical knowledge linking high blood pressure to an increased risk of nosebleeds (18).

While the present study identified some deficiencies of epistaxis awareness, it is crucial to acknowledge that there is some evidence of good epistaxis awareness in some aspects. According to the existing literature, the general public considers epistaxis to be a minor health concern (2, 16, 19). In contrast to our study, 72.7% of the participants considered epistaxis an emergency, which suggests a general awareness of the potential seriousness of epistaxis. This aligns with findings reported in another study (20). Furthermore, the studied sample suggests an acceptable level of parental preventative measures against epistaxis (e.g., local application of bacitracin or petrolatum ointment, digital trauma avoidance, and humidifying). Binary Logistics Regression was utilized to assess predictors of high parental literacy regarding epistaxis prevention strategies. A lower likelihood of demonstrating high literacy was observed in male parents, non-Saudi parents, and parents with higher educational attainment. These warrant further exploration and more comprehensive research is needed to investigate the association of predictive factors better.

According to our findings, only 47% of participants reported receiving first-aid training or awareness programs, which is consistent with other studies conducted in other cultural contexts as in Spain, approximately half of the respondents indicated they had received some first-aid training (21), a proportion similar to that found in a study carried out in Belgium (22), however, greater than that found in studies conducted in Turkey (23) and Greece (24). Therefore, it is imperative to disseminate first-aid knowledge widely through expanding access to first-aid training programs and incorporating basic first-aid information into public health campaigns to bridge the existing knowledge gap effectively. Our findings reveal varying levels of awareness among parents when asked about the appropriate time to consult an ENT specialist for epistaxis. Half of the participants correctly have recurrent nosebleeds that need prompt consultation. This suggests that many participants recognize the need for medical intervention when nosebleeds become a repeated concern, which is aligned with best practices in managing persistent symptoms (25).

Overall, these findings highlight the need for multifaceted educational interventions to address knowledge gaps, provide parents with accurate information regarding the management of epistaxis, and inform effective prevention strategies. Nora et al. conducted a quasi-experimental study to determine the effectiveness of epistaxis education guidelines in improving mothers’ performance in caring for children with epistaxis (9). The study concluded that, compared to the implementation level of the pre-educational guideline, mothers demonstrated significant improvement in their knowledge and attitude and reported practice scores in caring for epistaxis-affected children. Furthermore, by increasing parents’ awareness of basic management strategies, stress and intensity of this condition could be significantly reduced. Information sources vary in accuracy, completeness, and depth of coverage. Suboptimal information and outdated knowledge regarding epistaxis first aid can result in inappropriate interventions, potentially exacerbating the condition or hindering timely professional care. In order to determine the credibility and reliability of the information parents rely on to manage epistaxis, we surveyed parents about their sources of information about epistaxis first aid. Our findings indicate that the internet and social media were the predominant sources, followed by advice from friends and relatives and first aid courses, and a minority of respondents reported using Ministry of Health materials and books as their primary sources. These patterns align with those observed in studies by Saleem et al. (26) which aimed to investigate Saudi Arabians’ current understanding of epistaxis first-aid management and misconceptions, and Alam et al.’s (11) on parental knowledge of first-aid management of epistaxis in Taif, Saudi Arabia, in addition to Al-Shehri et al.’s (27) which explored the knowledge, attitude, and practice associated with first-aid management of epistaxis among the general population of Aseer, Saudi Arabia.

The limitations of the study include the self-reported nature of the research that presents as a significant limitation for the study since some parents may deliberately share inaccurate information due to looking up answers for their own questions from unreliable resources. Additionally, it might be necessary to assess their awareness by conducting interviews instead of distributing questionnaires for more cooperation and accuracy. It is recommended that a mixed-method approach should be utilized in future studies by quantitatively assessing parents’ knowledge using simulated scenarios, thus enhancing the accuracy of the results. To raise parents’ knowledge and awareness, we must focus on online initiatives and social campaigns so they can benefit from them.

## 5 Conclusion

This study highlights considerable knowledge gaps in epistaxis first aid among Saudi parents, emphasizing the need for targeted educational interventions. Despite a high exposure rate to epistaxis, misconceptions persist about crucial management steps, such as positioning and timing of nose pinching. Addressing these deficiencies through accessible training and standardized resources, especially in underserved regions and for demographic groups with lower literacy, can equip parents with accurate first-aid skills. Expanding community-based awareness campaigns, particularly on social media, could further enhance parental literacy and improve emergency responses to pediatric epistaxis.

## Data Availability

The original contributions presented in the study are included in the article/supplementary material, further inquiries can be directed to the corresponding author.
